# Mandibular clear cell odontogenic carcinoma

**DOI:** 10.1186/s12957-015-0693-4

**Published:** 2015-09-24

**Authors:** Ik Jae Kwon, Soung Min Kim, Emmanuel Kofi Amponsah, Hoon Myoung, Jong Ho Lee, Suk Keun Lee

**Affiliations:** Department of Oral and Maxillofacial Surgery, Dental Research Institute, School of Dentistry, Seoul National University, 101 Daehak-ro, Jongno-gu, Seoul, 110-768 Korea; Oral and Maxillofacial Microvascular Reconstruction LAB, Brong Ahafo Regional Hospital, Sunayni, Ghana; Department of Oral Pathology, College of Dentistry, Gangneung-Wonju National University, Gangneung, Korea

**Keywords:** Clear cell odontogenic carcinoma (CCOC), Fibular free flap, Mandibular reconstruction

## Abstract

**Background:**

Clear cell odontogenic carcinoma (CCOC) is a rare intraosseous carcinoma of the jaw; only 81 cases have been reported in the English literatures.

**Case presentation:**

We reported an additional case and reviewed the existing literature. A 70-year-old woman presented with a large painful radiolucent mandibular lesion from the right canine to the left angle area through the midline. No metastatic lymph nodes or distant metastases were detected. She underwent wide surgical resection and reconstruction with a composite fibula free flap. She had no recurrence or metastasis after 18 months.

**Conclusion:**

CCOC occurs predominantly in women in their 50s–70s in the mandible. Painless swelling is the most common symptom, followed by pain, teeth loosening, and paresthesia. CCOC has a good prognosis after surgery. In large mandibular CCOC, wide resection and composite fibula free flap reconstruction is the treatment of choice.

## Background

Clear cell odontogenic carcinoma (CCOC) is a rare intraosseous carcinoma of the jaw which was first described as a clear cell odontogenic tumor in 1985 by Hansen [1]. CCOC was initially known as clear cell odontogenic tumor or clear cell ameloblastoma. In 1992, CCOC was classified as odontogenic tumor by the WHO [2]; however, due to its aggressive tendency with local recurrence, regional lymph node metastasis, and distant metastasis [3], CCOC was considered to be a malignant tumor of odontogenic origin in the WHO classification of 2005 [4].

Only 81 cases have been reported in the English literatures to date excluding the present report [5–12]. CCOC occurs predominantly in the 5th to 7th decades in women in the mandible. Painless swelling is the most common symptom and pain, teeth loosening, and paresthesia follow. In this study, we reported an additional case and reviewed the existing literatures. On review of previous studies, our case was a rare large case, extending from the right canine area to the left angle across the midline. Simultaneous reconstruction with a microvascular fibula free flap was also rare among previous cases. The present study aims to report a rare CCOC case of a large lesion with free flap reconstruction and to review the previous literature.

## Case presentation

**Fig. 1:**
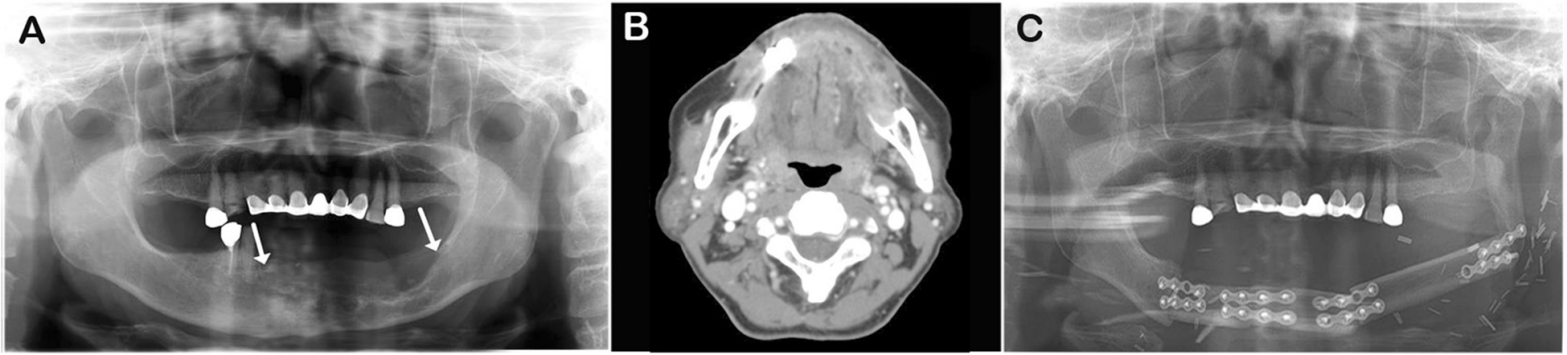
Pre- and post-operative radiographic findings. **a** A large radiolucent lesion from the right canine to the left angle in panorama (indicated by *arrows*). **b** CT image showing cortical and IAN canal destruction and involvement of mylohyoid and buccinator muscles. **c** Post-operative panoramic view, the fibula was successfully used to replace the mandible

A 70-year-old woman presenting with a large and painful radiolucent mandibular lesion was referred to the Department of Oral and Maxillofacial Surgery at Seoul National University Dental Hospital, Seoul, Korea. Her chief complaint was spontaneous pain, and she had a history of tooth extraction due to local pain in a private clinic. After extraction, she had no improvement in her symptoms. Radiological examination showed a large radiolucent mandibular lesion extending from the right canine to the left angle area through the midline (Fig. 1a). On enhanced computed tomography (CT) and magnetic resonance images (MRI), the inferior alveolar nerve canal was destroyed and the mylohyoid muscles and buccinator muscles were involved (Fig. 1b). No metastatic lymph node or distant metastasis was detected. Incisional biopsy, previously done at an outside hospital, resulted in undifferentiated carcinoma; however, the Department of Oral and Maxillofacial Pathology in our hospital diagnosed clear cell odontogenic carcinoma.

**Fig. 2:**
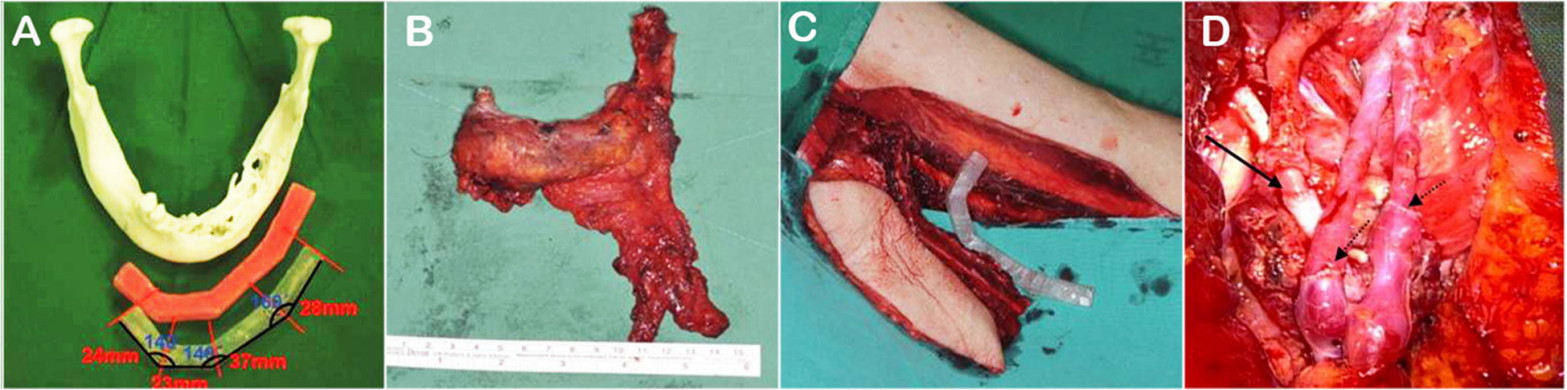
Intraoperative procedures. **a** The total required bone length was 115 mm, and a prefabricated resin stent was made. **b** The mandible and neck mass were removed together en bloc. **c** Reconstruction with a microvascular fibula free flap was performed. **d** The peroneal artery was anastomosed with the facial artery via an end-to-end mode (indicated by a long arrow), and the vena comitans was anastomosed with a branch of the internal jugular vein (indicated by short arrows) under a microscope

Surgical resection and reconstruction with a composite fibula free flap were prepared thoroughly. The total required bone length was 115 mm, and a prefabricated resin stent was made (Fig. 2a). Partial mandibulectomy from the left sigmoid notch to the right second premolar and selective neck dissection (left level I, II, III, right level I, II) were done under general anesthesia. The mandible and neck mass were removed together en bloc (Fig. 2b) with simultaneous reconstruction with a microvascular fibula free flap (Fig. 2c); vessel anastomosis was done under a microscope. The peroneal artery was anastomosed with the facial artery, and two vena comitans were anastomosed with branches of the internal jugular vein via end-to-end mode (Fig. 2d). Elective tracheostomy was planned for safe post-operative care.

**Fig. 3:**
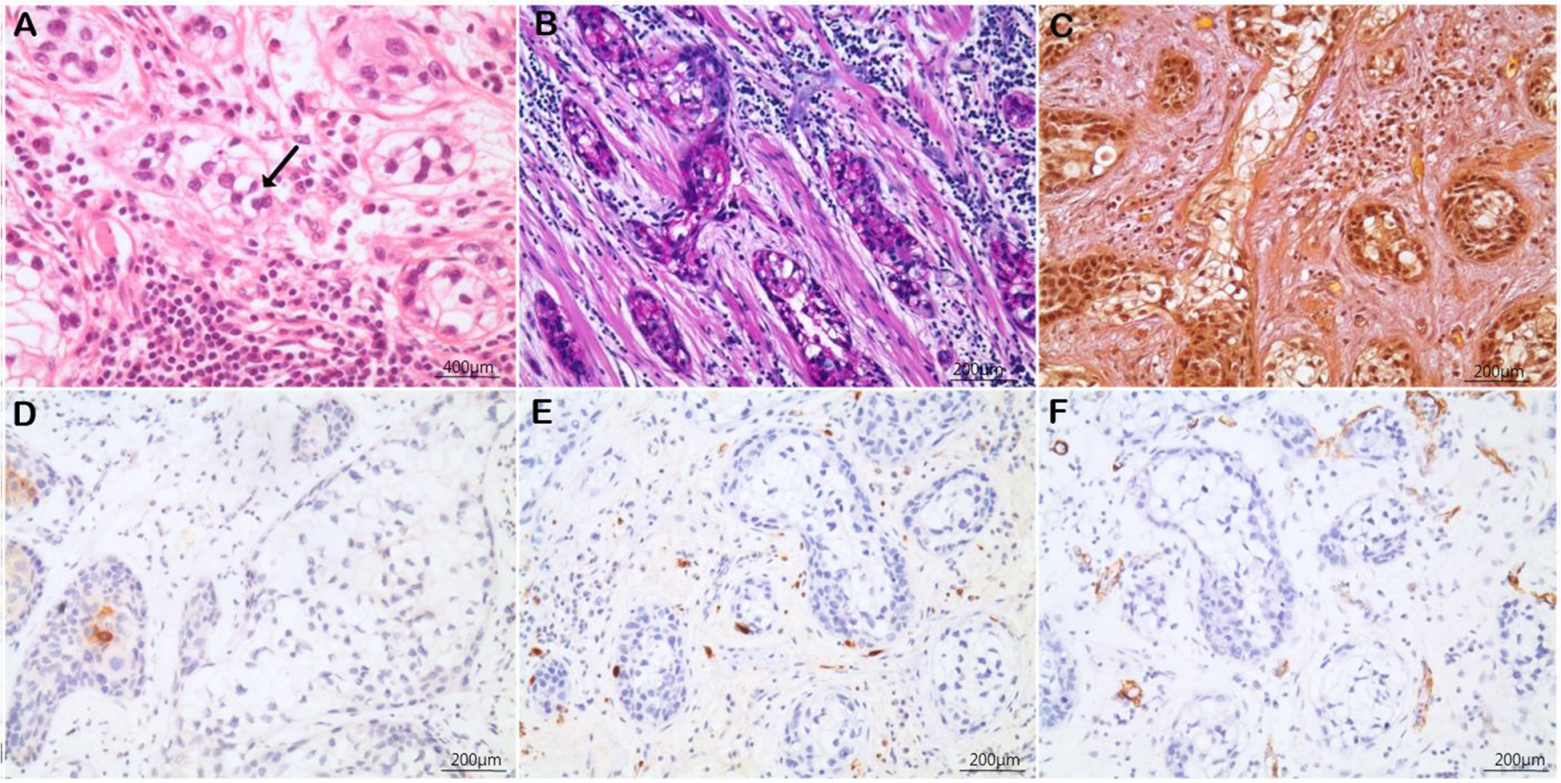
Histological findings. **a** Sheets and islands of vacuolated clear cells oval or polyhedral in shape with small dark-staining eccentric nuclei (indicated by an arrow). **b** PAS(+) identified glycogen positive that indicates clear cells. **c** Mucicarmine was negative. **d** CK-7 was focal positive. **e** S-100 was negative. **f** SMA was negative

The resected masses contained a diffusely infiltrative invasive tumor with no margin involvement. There were no metastatic cervical lymph nodes in the dissected mass. Perineural infiltration and vascular invasion were not seen. The final pathologic staging was pT4aN0M0 stage IVA. The tumor was composed of sheets and islands of vacuolated clear cells that were oval or polyhedral in shape with small dark-staining eccentric nuclei (Fig. 3a). Biphasic pattern and bone invasion could be seen. Inflammatory cells were observed around the tumor.

On immunohistochemical staining, PAS showed glycogen positive, indicating clear cells (Fig. 3b). Mucicarmine was negative, eliminating mucoepidermoid carcinoma (Fig. 3c). Expression of CK-7, which is seen in the majority of cases of carcinoma, was positive focally (Fig. 3d). In addition, S-100 was negative, ruling out melanoma (Fig. 3e). SMA, a marker of proliferation of periendothelial smooth muscle cells and myofibroblasts, was also negative (Fig. 3f). Because of the consistency with clear cells and other immunohistochemical results, the final diagnosis of CCOC was established.

During 18 months of follow-up, the patient had no recurrence and distant metastasis. Under the institutional review board (IRB) granted approval of Seoul National University Dental Hospital, the fibula was successfully replaced as the mandible and a symmetrical facial outline was confirmed in the panoramic view (Fig. 1c). Furthermore, the patient looked similar to her pre-operative state and did normal ambulation.

### Discussion

According to a review of the English literatures, a total of 81 CCOC cases were identified up to date. Since Zhang et al. [5] reported 6 cases and reviewed 67 cases, additional 8 cases were reported in the English literatures [6–12]. Thus, we reviewed total 81 CCOC cases and made a descriptive summary of our mini reviews in Table 1. CCOC has a female predilection, with an M/F ratio of 1:2 and a mean age of 55 (from 14 to 89). In addition, the majority of cases were located in the mandible with a Mn:Mx ratio of 3:1. The most common clinical symptom was swelling, followed by pain and paresthesia. The classic clinical presentation of CCOC has been reported to be of a painless swelling in the mandible or maxilla. In our case, the clinical symptoms were quite different from previous cases. The patient had a painful lesion, but there was no swelling. Because of the absence of swelling, the patient was misdiagnosed as a toothache in a private clinic before presenting to our hospital. In addition, this case was relatively rare in terms of large size and simultaneous reconstruction with a microvascular free flap.

**Table 1:** A mini review of English literatures with 81 CCOC cases

Categories	Parameters	No	Percentage
Age	Avg (min–max)	55 (14–89)	
Sex	Female	54	66.7 %
	Male	27	33.3 %
Location	Mandible	60	74.1 %
	Maxilla	21	25.9 %
Radiologic findings	Radiolucent	65	80.2 %
	Mixed	4	4.9 %
Signs and symptoms	Swelling	46	56.8 %
	Pain	16	19.8 %
	Teeth mobility	14	17.3 %
	Paresthesia	7	8.6 %
Treatment	Resection without ND	47	58.0 %
	Resection with ND	13	16.0 %
	Curettage or enucleation	15	18.5 %
Adjuvant therapy	Radiotherapy	14	17.3 %
	Chemotherapy	1	1.2 %

*ND* neck dissection

In some cases, CCOC was reported as difficult to diagnose. Kim et al. [12] reported a case of a well-defined unicystic radiolucent lesion that was comparable with a cystic lesion. At first, it was misdiagnosed as an infected cyst. In our mini review of the last 81 cases, the most frequent radiologic type was radiolucent (only 4 cases were mixed type). Thus, the possibility of misdiagnosis is relatively high, and the lesion could undergo decompression or curettage before pathologic examination. A radiolucent lesion with jaw enlargement and loosening teeth should be considered to possibly be malignant CCOC in order to identify and treat patients appropriately.

CCOC is also difficult to diagnose histopathologically. The differential diagnosis of jaw tumors with prominent cytoplasmic clearing includes intraosseous salivary gland tumors (epithelial-myoepithelial carcinoma) and metastatic tumors (clear cell renal cell carcinoma). Other odontogenic tumors may also show clearing of their constituent cells. Such tumors include calcifying epithelial odontogenic tumor and clear cell ameloblastoma. While the former is identified by the presence of psammomatous calcifications and amyloid deposits, the latter may be difficult to distinguish from CCOC [13]. In fact, some authors thought that clear cell ameloblastomas and CCOCs might represent a clinicopathological continuum of a single neoplastic entity [14]. In addition, clear cell carcinoma and CCOC are difficult, and in some cases, impossible to distinguish morphologically and immunohistochemically, despite a different cell of origin. Bilodeau et al. [15] suggested that location is the most important distinguishing criterion for these tumors.

In CCOC, surgical resection with a wide margin is the treatment of choice. Thus, proper jaw reconstruction is important and should be performed simultaneously with resection. Fibular free flap reconstruction is necessary when the resected jaw defect is large in the mandible; it provides several advantages over other donor sites, including adequate bone length, ease of graft dissection and contouring, a two-team approach, long pedicles with proper vessels, and minimal donor site morbidity. In this case, we obtained an adequate bone length (115 mm) and were able to reconstruct the mandible with satisfactory esthetics and no complications.

## Conclusions

Our survey of the English literature demonstrates that CCOC occurs to 5th to 7th decades in women in the mandible with painless swelling. In this case, the patient had a different symptom such as a painful toothache without swelling. We also found that it has a good prognosis after surgery. Radiographic images of CCOC generally demonstrate radiolucency but occasionally they are mixed. The differential diagnosis is broad, so a careful approach is necessary both clinically and immunohistochemically. In a large CCOC in mandible cases, wide resection and composite fibula free flap reconstruction is the treatment of choice.

## Consent

Written informed consent was obtained from the patient for publication of this manuscript and any accompanying images. A copy of the written consent is available for review by the Editor-in-Chief of this journal.
